# Hypoglycaemia, chronic kidney disease and death in type 2 diabetes: the Hong Kong diabetes registry

**DOI:** 10.1186/1472-6823-14-48

**Published:** 2014-06-13

**Authors:** Alice PS Kong, Xilin Yang, Andrea Luk, Kitty KT Cheung, Ronald CW Ma, Wing Yee So, Chung Shun Ho, Michael HM Chan, Risa Ozaki, Chun Chung Chow, Nicola Brown, Juliana CN Chan

**Affiliations:** 1 Department of Medicine and Therapeutics, The Chinese University of Hong Kong, Prince of Wales Hospital, Hong Kong, SAR, China; 2 Department of Epidemiology and Biostatistics, School of Public Health, Tianjin Medical University, Tianjin, China; 3 Asia Diabetes Foundation, Shatin, China; 4 Department of Chemical Pathology, Shatin, China; 5 Li KaShing Institute of Health Sciences, Shatin, China; 6 Hong Kong Institute of Diabetes and Obesity, The Chinese University of Hong Kong, Prince of Wales Hospital, Hong Kong, SAR, China

## Abstract

**Background:**

In patients with type 2 diabetes, chronic kidney disease (CKD) is associated with increased risk of hypoglycaemia and death. Yet, it remains uncertain whether hypoglycaemia-associated mortality is modified by CKD.

**Methods:**

Type 2 diabetic patients, with or without CKD at enrolment were observed between 1995 and 2007, and followed up till 2009 at hospital medical clinics. We used additive interaction, estimated by relative excess risk due to interaction (RERI) and attributable proportion due to interaction (AP) to examine possible synergistic effects between CKD and severe hypoglycaemia (defined as hospitalisations due to hypoglycaemia in the 12 months prior to enrolment) on the risk of death.

**Results:**

In this cohort of 8,767 type 2 diabetic patients [median age: 58 (interquartile range: 48 to 68) years; disease duration: 5 (1 to 11) years, men: 47.0%], 1,070 (12.2%) had died during a median follow-up period of 6.66 years (3.42-10.36) with 60,379 person-years.Upon enrolment, 209 patients had severe hypoglycaemia and 194 developed severe hypoglycaemia during follow-up (15 patients had both). In multivariable analysis and using patients without severe hypoglycaemia nor CKD as the referent group (683 deaths in 7,598 patients), severe hypoglycaemia alone (61 deaths in 272 patients) or CKD alone (267 death in 781 patients) were associated with increased risk of death [Hazard ratio, HR: 1.81(95%CI: 1.38 to 2.37) and 1.63 (1.38 to 1.93) respectively]. Having both risk factors (59 deaths in 116 patients) greatly enhanced the HR of death to 3.91 (2.93 to 5.21) with significant interaction (RERI: 1.46 and AP: 0.37, both p-values < 0.05).

**Conclusions:**

Severe hypoglycaemia and CKD interact to increase risk of death in type 2 diabetes patients.

## Background

When glycaemic control is targeted to a glycated haemoglobin (HbA_1c_) of 7% in patients suffering from type 2 diabetes, microvascular and macrovascular complications and all-cause mortality is reduced [1]. Large scale randomized controlled trials including the ADVANCE (Action in Diabetes and Vascular disease: preterAx and diamicroN modified release Controlled Evaluation) [2], ACCORD (Action to Control Cardiovascular Risk in Diabetes) [3] and VADT (Veteran Affairs Diabetes Trial) [4] have been conducted to determine whether intensive lowering of HbA_1c_ to less than 7% would further improve cardiovascular outcomes [2-4]. However, the results from the ACCORD study rekindled the debate on risk-benefit ratio of these intensive strategies with the premature discontinuation of the intensive blood-glucose lowering arm in the ACCORD Study [3,5,6]. Subsequent analysis of both the ACCORD and ADVANCE Study revealed that while intensive glycaemic control increased the risk of severe hypoglycaemia which was associated with increased risk of death, the hazard ratios (HR) were in fact lower in the intensively-treated group, suggesting that intensive monitoring with prompt corrective actions might mitigate the potential harm of intensive blood glucose lowering [6,7].

However, due to volunteer effects and to the unique environment of a randomized controlled trial setting, the applicability of these findings to a real world setting remains uncertain. To date, there is a paucity of long-term data with adequate documentation of confounders to allow detailed analysis of the causes and consequences of hypoglycaemia in type 2 diabetes [8]. To this end, renal dysfunction is a potential risk amplifier for death associated with hypoglycaemia. Microalbuminuria is known to be the earliest clinical evidence of diabetic nephropathy and up to 20-40% of diabetic patients progress to overt nephropathy if left untreated [9]. In Asia, micro- and macroalbuminuria are common in type 2 diabetic patients, affecting 50-60% of patients with type 2 diabetes [10] while about 16% of type 2 diabetic patients suffer from chronic kidney disease (CKD) [11]. Given the high rates of diabetic nephropathy (usually considered as nephropathy with or without CKD) and CKD (based on eGFR alone) in Asian populations and the high risk of hypoglycaemia and clinical outcomes in CKD patients, we questioned whether the co-occurrence of these conditions will further increase the risk of future events. In a prospective registry with detailed documentation of risk factors, complications, drug use and clinical outcomes, we explored the prognostic significance of CKD on incident severe hypoglycaemia and the interactive effects of prevalent CKD and severe hypoglycaemia on future risk of clinical outcomes.

## Methods

We retrospectively analysed the data of a prospective observational study of type 2 diabetic patients enrolled into the Hong Kong Diabetes Registry. Upon enrolment, diabetic patients undergo comprehensive assessments which follow a structured protocol whose methodology has been described previously [11-14]. Briefly, the Hong Kong Diabetes Registry was established in 1995 at the Prince of Wales Hospital, which serves a population of over 1.2 million. Since 1995, diabetic patients attending medical clinics at the Prince of Wales Hospital can be referred to the Diabetes Centre for comprehensive assessment based on the European DIABCARE protocol [15]. Hong Kong has a heavily subsidized healthcare system, so the vast majority of patients with chronic illnesses, including diabetes, are managed in public hospitals governed by the Hospital Authority (HA), which provide for 95% of the total hospital bed-days in Hong Kong [12]. Once the participants are entered into the Registry, their outcomes including hospital admissions will be monitored until the death of the patient [11-14]. From 1995 to the 31^st^ December 2007, 10,129 patients have been enrolled into the cohort. After excluding 417 patients with type 1 diabetes (including missing data on classification of diabetes type), and 945 with missing variables used in the analysis, 8,767 patients were included in this analysis.

The enrolled patients periodically underwent a comprehensive 4-hour assessment for quality assurance including interview by diabetes nurses, anthropometric measurements, blood and urine tests, fundus examination and podiatry assessment. After 8 hours of overnight fast, blood was sampled for assay of fasting plasma lipids [total cholesterol (TC), HDL-C, triglyceride (TG) and calculated LDL-C], glucose, HbA_1c_, renal and liver function tests. We used spot urinary albumin: creatinine ratio (ACR) to define albuminuria (ACR ≥ 2.5 mg/mmol in men and ≥3.5 mg/mmol in women). The abbreviated Modification of Diet in Renal Disease Study (MDRD) formula recalibrated for Chinese [16] was used to define CKD as eGFR < 60 ml/min/1.73 m^2^[17]. All laboratory analyses were performed using standard methods in the Department of Chemical Pathology of the Prince of Wales Hospital. The laboratory is accredited by the Australian National Association of Testing Authorities. Informed written consent was obtained from all participants and the study was approved by the Chinese University of Hong Kong Clinical Research Ethics Committee prior to its initiation.

Severe hypoglycaemia was defined as one or more hospitalisations due to hypoglycaemia 12 months prior to enrolment (‘at enrollment’), in order not to miss events and subjects with characteristics relevant to this present analysis, or during the follow-up period [14], as defined from enrolment to death or 31^st^ January, 2009. Using the same definintion for severe hypoglycaemia, we had previously reported that severe hypoglycaemia identified vulnerable type 2 diabetic patients who were at risk for premature death and were associated with cancer subphenotypes [14]. We ascertained all clinical outcomes using the HA Central Computer Management System (CMS), which records diagnoses of all hospital discharges, including mortality based on the International Classification of Diseases, Ninth Revision (ICD-9). The mortality data was cross-checked with the Hong Kong Death Registry and the cause of death was defined by the principal discharge diagnosis.

### Statistical analysis

The Statistical Analysis System (Release 9.30) was used to perform all analyses (SAS Institute Inc., Cary, NC, USA) unless specified. Follow-up time was calculated as the period in years from the first enrolment to the date of death or 31^st^ January 2009, whichever came first. All data were expressed as mean ± SD or median (interquartile range, IQR). Cox proportional hazard regression was used to obtain hazard ratio (HR) and 95% confidence interval (CI) of variables of interest. We used the Yes/No coding scheme for all major drug use at enrolment.

Immortal time is defined as the period of time without exposure to hypoglycemia from the point of enrolment into the study to the date of first hospitalization due to hypoglycemia during the follow-up period in this study. This however, may introduce immortal time bias [18,19]. In this regard, we performed a validation study of various methods to cope with immortal time bias and found that removal of immortal time led to the least inflated hazard ratio [20]. Thus, in this study, we excluded 896.8 person-years of immortal time from the analysis by moving the commencement point of follow-up from date of enrolment to the time of first hospitalization due to hypoglycemia during follow-up. For patients with severe hypoglycemia at enrolment, the immortal period was considered ‘0’ and their clinical profile at enrolment was used for analysis. For patients who developed severe hypoglycemia during follow-up, the enrolment was moved to the time of severe hypoglycemia during follow-up. As the metabolic profile may deteriorate over time, as in our validation study [20], we used multivariable linear regression to obtain partial regression coefficients of age (β_a_) and duration of diabetes (β_b_) from all other covariables at enrolment and used the estimated values of HbA_1c_, body mass index (BMI), systolic blood pressure (SBP), diastolic blood pressure (DBP), LDL-C, HDL-C, TG, ACR, and eGFR derived from the following formulae: X_t_ = X_b_ + β_a_ T_i_ + β_b_ T_i_, where X_t_ is the value at hypoglycemia during follow-up, X_b_ is the value at baseline, and T_i_ is the immortal time (Additional file 1: Table S1 for β_a_ and β_b_). Prior cardiovascular diseases (CVD) and cancer were re-estimated taking into consideration whether these events occurred at enrolment, or during the immortal time period.

We further used relative excess risk due to interaction (RERI) and attributable proportion due to interaction (AP) [18,21] to estimate additive interaction between hypoglycemia and CKD on all-cause death. The RERI is the excess risk due to interaction relative to the risk without exposure. AP refers to the attributable proportion of disease due to interaction in persons with both exposures. RERI >0 or AP > 0 indicates significant additive interaction.

A three-step adjustment scheme was used to control for covariables. First, we obtained the HR in univariable analysis, followed by further adjustment for age, sex, use of tobacco and alcohol, BMI, duration of diabetes, HbA_1c_, systolic BP, LDL-C, HDL-C, TG, natural log-transformed (spot urinary ACR + 1), eGFR, prior CVD and/or cancer at enrolment and drug use at baseline.

The plots of LOG [-LOG (Survival function)] versus LOG (follow-up time in years) were used to check proportional hazards assumption for categorical variables, while the Supremum test was used to check the assumption for continuous variables [19]. In case of violation of the proportional hazard assumption, a stratified Cox model analysis on the variable concerned was used to adjust for its confounding effect. To avoid co-linearity, SBP but not DBP, and BMI but not waist circumference, were used in the model fitting. Pearson correlation was used to exclude highly correlated variables from the models (correlation coefficient > 0.60) [22]. A p value <0.05 (two-sided) was considered statistically significant.

The Statistical Package for Social Sciences version 16 (SPSS, Chicago, US) was used to obtain the adjusted plot of cumulative mortality stratified by hypoglycemia and CKD and their combination over time.

## Results

The median age of the cohort was 58 years (IQR:48–68) with a disease duration of 5 [1-11] years. During a median follow-up period of 6.66 (3.42-10.36) years with 60,379 person-years, 1,070 patients had died with a mortality rate of 17.7 (16.7-18.8) per 1000-person-years. The deceased were older, more likely to be men, ex/current smokers and alcohol drinkers, had longer disease duration, higher SBP, HbA_1c_, LDL-C and lower BMI and HDL-C than those who survived. They were also more likely to have history of CVD and cancers (all p < 0.05), poorer renal function (higher ACR, lower eGFR) and more frequent severe hypoglycaemia events (both at enrolment and during follow-up) (p < 0.0001) (Table 1).

**Table 1:** Clinical and biochemical characteristics of the study cohort

	**Patients who survived (n = 7697)**	**Patients who died (n = 1070)**	
	Median (25th to 75th) or n(%)	Median (25th to 75th) or n(%)	P value†
Age, years	57(47–66)	69(62–75)	<0.0001
Male gender	3586(46.6%)	533(49.8%)	0.0477
Smoking status			<0.0001
Ex-smoker	1094(14.2%)	256(23.9%)	
Current smoker	1310(17.0%)	197(18.4%)	
Alcohol intake			<0.0001
Ex-drinker	846(11.0%)	206(19.3%)	
Current drinker	720(9.4%)	57(19.3%)	
Body mass index, kg/m^2^	24.9(22.6-27.6)	24.2(21.8-26.6)	<0.0001
Duration of diabetes, years	5(1-10)	9(4–15)	<0.0001
Systolic BP, mmHg	132(120–145)	144(128–159)	<0.0001
Diastolic BP, mmHg	75(68–82)	75(68–83)	0.4583
HbA_1c_, %	7.2(6.3-8.4)	7.5(6.5-8.9)	<0.0001
LDL-C, mmol/L	2.98(2.40-3.60)	3.14(2.50-3.90)	<0.0001
HDL-C, mmol/L	1.28(1.09-1.52)	1.25(1.03-1.54)	0.0108
Triglyceride, mmol/L	1.37(0.97-1.99)	1.36(0.98-2.00)	0.6668
Spot urinary ACR, mg/mmol	1.69(2.72)	13.42(2.06-120.20)	<0.0001
Prior cardiovascular disease	972(12.6%)	321(30.0%)	<0.0001
Prior cancer	193(2.9%)	69(6.5%)	<0.0001
**Medication use at enrollment**			
Renin-angiotensin system inhibitors	1687(21.9%)	306(28.6%)	<0.0001
Lipid lowering drugs	1509(19.6%)	170(15.9%)	0.0038
Oral anti-diabetes drugs	5335(69.3%)	626(58.5%)	<0.0001
Insulin	1197(15.6%)	311(29.1%)	<0.0001
**CKD events at baseline**			
eGFR, ml min^-1^ 1.73 m^-2^ at baseline	105.6(86.2-126.0)	79.0(51.7-104.7)	<0.0001
eGFR < 60 ml min^-1^ 1.73 m^-2^ at baseline	571(7.4%)	326(30.5%)	<0.0001
**Major hyopglycaemia events**			
12 months prior to enrollment	146(1.9%)	63(5.9%)	<0.0001
During follow-up only§	122(1.6%)	57(5.3%)	<0.0001
Either of them	268(3.5%)	120(11.2%)	<0.001

Abbreviations: HbA_1c_, glycated haemglobin; LDL-C, low-density lipoprotein cholesterol; HDL-C, high-density lipoprotein cholesterol; BP, blood pressure; ACR, albumin:creatinine ratio; eGFR, estimated glomerular filtration rate;

†, Derived from Wilcoxon Two-Sample test or Chi-squared test where appropriate;

§, Defined as hypoglycaemia events that required hospitalisations. For patients who developed the event during follow-up, the time period from enrollment to the time of hospitalisation was removed as the immortal time period so that the follow-up started at the time of admission for hypoglycaemia. In these patients, all baseline values of continuous covariables and prior cancer and cardiovascular disease were re-estimated by treating the time of admission for hypoglycaemia as the enrollment date.

After excluding 209 patients who had documented hypoglycemia upon enrollment, 179 patients developed hypoglycemia with an event rate of 3.02(95%CI: 2.57-3.46) per 1,000 person-years. In this cohort, 897 patients (10.2%) had CKD at enrollment, amongst whom, 116(12.9%) had hospitalizations due to hypoglycemia (50 at enrollment, 60 during follow-up and 6 had events both at enrollment and during follow-up). In patients without CKD (n = 7870), 272(3.46%) had hospitalizations due to hypoglycemia (144 at enrollment, 119 during follow-up and 9 with events both at enrollment and during follow up) with higher rate of hypoglycemia in patients with CKD than those without (p < 0.0001).

**Figure 1:**
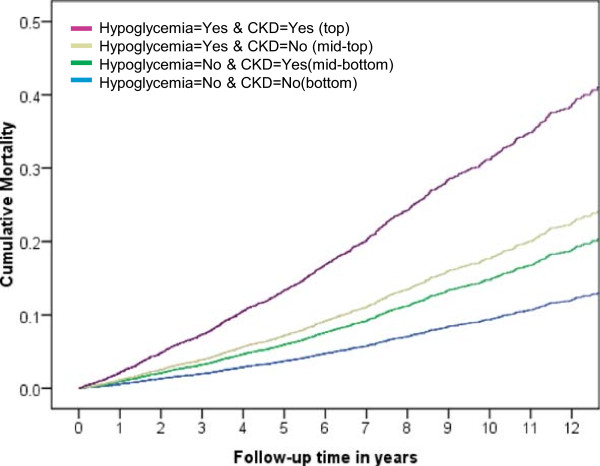
**Cumulative incidence of all-cause death stratified by the presence of chronic kidney disease (CKD) and severe hypoglycaemia.** Legend: The model adjusted for age, sex, body mass index (BMI), smoking status, alcohol use , low-density lipoprotein cholesterol (LDL-C), high density-lipoprotein cholesterol (HDL-C), triglyceride (TG), systolic blood pressure (SBP), HBA_1c_, duration of disease, and log (urinary albumin to creatinine ratio [ACR] +1), prior history of cardiovascular disease and cancer as well as drug use at enrollment, including lipid lowering drugs, renin-angiotensin system inhibitors, oral anti-diabetic drugs and insulin; P < 0.0001.

In multivariable analysis, severe hypoglycemia predicted mortality among patients with CKD with a HR of 2.63(1.95 to 3.55) and to a lesser extent, among those without CKD [1.72(1.31 to 2.26)] (Table 2). After adjusting for confounders, CKD enhanced the HR of severe hypoglycemia for all-cause mortality from 1.81(1.38 to 2.37) to 3.91(2.93 to 5.21) (Table 3) with significant additive interaction between hypoglycemia and CKD (RERI: 1.46 (0.31 to 2.61, p = 0.0126); AP: 0.37(0.17 to 0.58, p = 0.0177) (Additional file 2: Table S2). From 1 December 1996 to 30 July 2005, 52.7% (n = 4618) of the diabetic patients have documented use of sulfonylurea and after further adjustment for the use of sulfonylurea during this period, this had little impact on the HRs.Figure 1 shows the multivariable cumulative mortality of patients categorized by hypoglycemia and CKD with the highest mortality rate in those with both CKD and hypoglycemia.

**Table 2:** Hazard ratios of severe hypoglycaemia and chronic kidney disease (CKD) for the risk of all-cause death in patients with type 2 diabetes

**Exposures**	**Number of death (%)**	**Hazard ratio**	**95% CI**	**P value**
**Independent Models**				
Patients without CKD				
Model 1: Hypoglycemia	749(9.45%)	3.48	2.68 to 4.52	<0.0001
Model 2: Hypoglycemia	749(9.45%)	1.81	1.38 to 2.38	<0.0001
Model 3: Hypoglycemia	749(9.45%)	1.72	1.31 to 2.26	<0.0001
Patients with CKD				
Model 1: Hypoglycemia	326(36.34%)	2.87	2.16 to 3.82	<0.0001
Model 2: Hypoglycemia	326(36.34%)	2.70	2.00 to 3.64	<0.0001
Model 3: Hypoglycemia	326(36.34%)	2.63	1.95 to 3.55	<0.0001
**Interactive Models**				
Model 1				
Hypoglycemia = Yes and CKD = No	61(22.43%)	3.47	2.67 to 4.52	<0.0001
Hypoglycemia = No and CKD = Yes	267(34.19%)	5.70	4.93 to 6.57	<0.0001
Hypoglycemia = Yes and CKD = Yes	59(50.86%)	16.21	12.38 to 21.23	<0.0001
Hypoglycemia = No and CKD = No	683(8.99%)		Reference	
Model 2				
Hypoglycemia = Yes and CKD = No	61(22.43%)	1.87	1.43 to 2.44	<0.0001
Hypoglycemia = No and CKD = Yes	267(34.19%)	1.75	1.49 to 2.07	<0.0001
Hypoglycemia = Yes and CKD = Yes	59(50.86%)	4.32	3.25 to 5.75	<0.0001
Hypoglycemia = No and CKD = No	683(8.99%)		Reference	
Model 3				
Hypoglycemia = Yes and CKD = No	61(22.43%)	1.81	1.38 to 2.37	<0.0001
Hypoglycemia = No and CKD = Yes	267(34.19%)	1.63	1.38 to 1.93	<0.0001
Hypoglycemia = Yes and CKD = Yes	59(50.86%)	3.91	2.93 to 5.21	<0.0001
Hypoglycemia = No and CKD = No	683(8.99%)		Reference	

Model 1, not adjusted for other covariables at enrollment;

Model 2, adjusted for age, sex, body mass index (BMI), smoking status, alcohol use, low-density lipoprotein cholesterol (LDL-C), high density-lipoprotein cholesterol, triglyceride, systolic blood pressure (SBP), HBA_1c_, duration of disease, and Ln (urinary albumin to creatinine ratio [ACR] +1), prior cardiovascular disease and prior cancer. As BMI violated the proportional hazard assumption, Cox models stratified on quartiles of BMI were used to adjust for the confounding effect of BMI;

Model 3, further adjusted for drug use at enrollment, including lipid lowering drugs, renin-angiotensin system inhibitors, oral anti-diabetes drugs and insulin.

**Table 3:** **Further adjustment for the use of sulfonylurea from 1 December 1996 to 30 July 2005 in model 3 of Tables**2**, 3**

**Exposures**	**Number of death (%)**	**Hazard ratio**	**95% CI**	**P value**
**Independent Models**				
Patients without CKD				
Hypoglycemia	749(9.45%)	1.72	1.31 to 2.26	<0.0001
Patients with CKD				
Hypoglycemia	326(36.34%)	2.64	1.96 to 3.56	<0.0001
**Interactive Models**				
Hypoglycemia = Yes and CKD = No	61(22.43%)	1.81	1.38 to 2.37	<0.0001
Hypoglycemia = No and CKD = Yes	267(34.19%)	1.63	1.38 to 1.93	<0.0001
Hypoglycemia = Yes and CKD = Yes	59(50.86%)	3.91	2.93 to 5.21	<0.0001
Hypoglycemia = No and CKD = No	683(8.99%)		Reference	

Covariables adjusted were listed in model 3 of Table 2 and ever use of sulfonylurea from 1 December 1996 to 30 July 2005.

Among the 1,070 deaths, the major causes were cancer (23.4%, n = 250), CVD (22.9%, n = 245), respiratory disease (15.1%, n = 162) and renal disease (13.6%, n = 146). After adjustment for confounders, CKD was an independent predictor for cardiovascular [1.54 (1.20 to 2.13)] related deaths, whereas severe hypoglycaemia was an independent predictor for renal [3.10 (1.97 to 4.89)] and cancer-related deaths [1.97 (1.26 to 3.07)] (Table 4).

**Table 4:** Hazard ratios of severe hypoglycaemia events and chronic kidney disease (CKD) for the risk of cause-specific death in patients with type 2 diabetes

**Causes of death**	**Number of death (%)**	**Hazard ratio**	**95% CI**	**P value**
**Cardiovascular disease**				
Model 1				
Hypoglycemia events	14(3.61%)	1.48	0.86 to 2.54	0.1619
CKD	83(9.25%)	7.15	5.45 to 9.38	<0.0001
Model 2				
Hypoglycemia events		0.99	0.57 to 1.71	0.9603
CKD		1.63	1.19 to 2.23	0.0021
Model 3				
Hypoglycemia events		0.99	0.57 to 1.72	0.9790
CKD		1.54	1.20 to 2.13	0.0081
**Renal disease**				
Model 1				
Hypoglycemia events	26(6.70%)	4.27	2.76 to 6.61	<0.0001
CKD	88(9.81%)	20.92	14.79 to 29.58	<0.0001
Model 2				
Hypoglycemia events		3.20	2.03 to 5.02	<0.0001
CKD		3.47	2.35 to 5.15	<0.0001
Model 3				
Hypoglycemia events		3.10	1.97 to 4.89	<0.0001
CKD		3.42	2.29 to 5.11	<0.0001
**Respiratory disease**				
Model 1				
Hypoglycemia events	13(3.35%)	2.29	1.29 to 4.07	0.0048
CKD	45(5.02%)	5.19	3.65 to 7.39	<0.0001
Model 2				
Hypoglycemia events		1.24	0.69 to 2.23	0.4741
CKD		1.54	1.03 to 2.31	0.0358
Model 3				
Hypoglycemia events		1.21	0.67 to 2.18	0.5235
CKD		1.44	0.95 to 2.17	0.0845
**All-site cancer**				
Model 1:				
Hypoglycemia events	24(6.19%)	3.21	2.10 to 4.92	<0.0001
CKD	28(3.12%)	1.39	0.94 to 2.08	0.1030
Model 2				
Hypoglycemia events		2.09	1.34 to 3.26	0.0011
CKD		1.00	0.64 to 1.54	0.9805
Model 3				
Hypoglycemia events		1.97	1.26 to 3.07	0.0027
CKD		0.92	0.59 to 1.44	0.7192

Adjustment schemes for models 1 to 3 were the same as listed in Tables 2 and 3.

## Discussion

To our knowledge, this study is the first report on the increased risk of all-cause mortality in Chinese type 2 diabetic patients with co-occurrence of hypoglycaemia episodes and CKD in a real-world setting. Compared to patients without CKD or severe hypoglycaemia, the co-occurrence of these two risk factors markedly increased the risk of all-cause mortality by four-fold. While CKD predicted CVD-related deaths, hypoglycaemia predicted renal and cancer-related deaths.

The premature termination of ACCORD study has led to heated debates regarding the safety of tight glycaemic control and the impact of hypoglycaemia on mortality [5,6]. In the ACCORD Study, the all-cause mortality rate (mainly due to CVD) was 21% higher in the intensive treatment group (target HbA_1c_ < 6%) compared to the standard treatment group (target HbA_1c_ 7-7.9%) [3]. Although detailed post-hoc analysis did not reveal direct association between hypoglycaemia and CVD [6], these findings have alerted clinicians to the importance of individualizing treatment goals and strategiesin order to maximize benefits and minimize harm [23].

Hitherto, the risk-benefit ratios of intensive glycaemic control in type 2 diabetes have not been sufficiently studied [8]. While intensive glycaemic control may help to reduce the risks of development of diabetes-related complications, tight control of glycaemia inevitably leads to hypoglycaemia. In diabetic patients with advanced diseases, hypoglycaemia may amplify the adverse consequences of cognitive impairment, dementia, cardiac ischemia and arrhythmia [24,25]. In the United Kingdom Prospective Diabetes Study (UKPDS), the annual rate of major hypoglycaemia, defined by symptoms requiring assistance from a third-party or medical intervention, ranged from 0.7% in patients treated with oral drugs to 1.8% in the insulin-treated patients [26]. In the ADVANCE Study, during a median follow-up period of 4 years, 231 (2.1%) patients developed severe hypoglycaemia (defined as blood glucose < 2.8 mmol/l with neurological dysfunction and third-party assistance) which was associated with 2–3 fold increased risk of microvascular complications, cardiovascular events and all-causes mortality [7].

### Additive effects between CKD and hypoglycaemia on death

Our results, collected in a usual care setting, indicated that severe hypoglycaemia occurred in 4.4% of patients with type 2 diabetes with an annualized event rate of approximately 0.3%. After adjusting for covariables, hypoglycaemia markedly increased the risk of all-cause mortality, especially in those with CKD, which accounted for 10.2% of the cohort. Besides malnutrition and reduced gluconeogenesis (approximately one fifth of plasma glucose originated from renal gluconeogenesis), diabetic patients with CKD often have altered drug metabolism and autonomic neuropathy with hypoglycaemic unawareness, which put them at high risk of hypoglycaemia [27]. In this context, renal dysfunction is a major risk factor for drug-induced hypoglycaemia [28]. In a 15-month retrospective study reported by our group [29], among 127 type 1 and type 2 diabetic patients hospitalized due to drug-induced hypoglycaemia (which accounted for 0.5% of the total medical admissions), factors includingold age, institutionalization and renal dysfunction predicted hypoglycaemia-associated death. Many of these hypoglycaemia events were related to the use of sulphonylureas. Other researchers have reported experimental and clinical data showing that hypoglycaemia might cause arrhythmia, abnormal haemostasis and neurohormonal dysregulation which substantially increase the risk of CVD [24].

Apart from common risk factors shared by CKD and CVD, changes in micro-environment associated with CKD can amplify CVD risk [30]. In a meta-analysis of 105,872 participants, from general population cohorts, both eGFR and albuminuria were independent predictors for all-cause and cardiovascular mortality [31]. The high CVD risk associated with CKD is multifactorial including anaemia, abnormal bone metabolism, vascular calcification, low grade inflammation and oxidative stress [32]. Using this Registry, we have reported the predictive values of reduced eGFR for micro- and macrovascular complications [11] as well as all-cause mortality [33]. These observational findings concur with the VADT Study, showing that renal dysfunction and macroalbuminuria also predict coronary heart diseases [4].

### Hypoglycaemia and all-cause deaths

In our analysis, type 2 diabetic patients who died before the censor date were more likely to use insulin and renin-angiotensin system (RAS) inhibitors. This pattern of drug usage was compatible with long disease duration, progressive beta cell failure and high cardiovascular-renal risk in these subjects. Since many patients were enrolled in the 1990’s when the beneficial effects resulted from the utility of these drugs was less well documented, the usage of RAS inhibitors and statin was relatively low which might have contributed to the high event rates in this cohort. In our previous analysis [12], we reported that urinary ACR, eGFR and insulin use predicted all-cause death. The combination of renal dysfunction and insulin use put patients at high risk for hypoglycaemia if they were not monitored closely. Of note, over 20% of our patients died of cancer, a proportion similar to CVD-related deaths. Thus, multiple organ dysfunction, abnormal metabolic milieu and malnutrition, often associated with CKD and cancer [34,35], might contribute to the additive interactions between CKD and hypoglycaemia on all-cause death in our cohort. In support of these findings, we also found that while CKD predicted CVD-related deaths, hypoglycaemia predicted cancer and renal-related deaths. Thus, despite the theoretical risk of hypoglycaemia for CVD, our study was only able to confirm the risk association of hypoglycaemia with all-cause but not CVD-related deaths, which is in agreement with the ACCORD study. Taken together, we argue that hypoglycaemia might be a marker of frailty and multiple organ dysfunction including cancer which should alert clinicians to reassess the clinical status and screen for silent conditions such as CKD and occult cancer. To this end, the 1.3-3 fold increased risk for multiple morbidities including cancer in diabetes is now well recognized [36,37] and support the frequent co-occurrence of hypoglycaemia, CKD and non-CVD deaths in our analysis.

### Limitations

Firstly, in this real-world dataset, all clinical outcomes were ascertained by ICD-9 codes, which might contain errors due to misclassification and/or under-coding. However, validation analysis suggested that the estimated predictive value of administrative data for clinical outcomes could be as high as 95% for acute myocardial infarction and stroke [38,39]. Secondly, we only included severe hypoglycaemic episodes requiring hospitalizations, which were specific but not sensitive indicators of these adverse events. Since our database did not systematically capture minor hypoglycaemia not requiring admission, under-estimation of true rates of hypoglycaemia was likely. Although our analysis did not confirm the risk association between severe hypoglycaemia and CVD-related deaths, it remained plausible that multiple minor hypoglycaemic episodes could have cumulative adverse effects on cardiovascular and cerebrovascular functions with direct or indirect impacts on mortality. Thirdly, we captured and adjusted the drug use at baseline but did not have data on changes of concomitant medications and metabolic profiles during the whole follow-up. Fourthly, only eGFR at baseline was used to estimate the occurrence of CKD events because we did not measure eGFR on a regular basis among all the patients in our cohort. Thus, some CKD events during follow-up had been missed.

## Conclusions

Using a real-world registry, we confirmed the independent and interactive effects of CKD and severe hypoglycaemia on all-cause death, with hypoglycaemia independently predicting cancer and renal-related death. These findings added to the growing body of literature on the vulnerability of diabetic patients with CKD and called for comprehensive assessment in patients with hypoglycaemia to screen for silent conditions such as CKD and cancer as well as review of treatment regimens to reduce the risk of hypoglycaemia in patients with these comorbidities.

### Ethical approval

The Joint Chinese University of Hong Kong and New Territories East Cluster Clinical Research Ethics Committee.

## Competing interests

We declare that we have no conflicts of interest except for JC, who has received honorarium from Bayer, Boehringer Ingelheim, Daiichi-Sankyo, Eli-Lilly, GlaxoSmithKline, Merck Sharp & Dohme, Merck Serono, Pfizer, Astra Zeneca, Sanofi, Novo-nordisk and/or Bristol-Myers Squibb for consultancy or delivery of lectures ; AK who has received honorarium for consultancy or delivery of lectures from Astra Zeneca, Novo-nordisk, Eli-Lilly, Merck Serono, Pfizer, Jassen, Sanofi and Nestle; and RM who has received honorarium for consultancy or delivery of lectures, from AstraZeneca, Boehringer Ingelheim, Danone, Eli Lilly, Nestle, Pfizer and Sanofi. The proceeds have been partially donated to the Chinese University of Hong Kong, American Diabetes Association and other charity organizations to support diabetes research and education. The Chinese University of Hong Kong has received research grants from the above companies. Other authors declared no conflict of interest with this manuscript.

## Authors’ contributions

AK, XY, RO, NB and JC contributed to the analysis and preparation of the manuscript. AK, AL, KC, RM, WS, CH, MC, RO, CC and JC contributed to the collection of the data. JC, the corresponding author, accepts full responsibility for the content of this paper. All authors read and approved the final manuscript.

## Pre-publication history

The pre-publication history for this paper can be accessed here:

http://www.biomedcentral.com/1472-6823/14/48/prepub

## Supplementary Material

Additional file 1: Table S1 Estimated partial regression coefficients of age and duration of diabetes for metabolic indicators at baseline. Click here for file

Additional file 2: Table S2 Additive interaction of hypoglycaemia events and chronic kidney disease for all-cause death in type 2 diabetes. Click here for file
