# Supplementary material for: Hypoglycaemia, chronic kidney disease and death in type 2 diabetes: the Hong Kong diabetes registry
Source: BMC Endocr Disord. 2014 Jun 13;14:48. doi: 10.1186/1472-6823-14-48 (PMC4151079; doi:10.1186/1472-6823-14-48)
Supplement: Additional file 1: Table S1 — Estimated partial regression coefficients of age and duration of diabetes for metabolic indicators at baseline. [file 1472-6823-14-48-S1.doc]

Additional file 1: Table S1 Estimated partial regression coefficients of age and duration of diabetes for metabolic indicators at baseline

| Variables | Partial regression coefficient of age | Partial regression coefficient of  duration of diabetes |
| --- | --- | --- |
| HbA1c | -0.01484 | -0.00024631 |
| BMI | -0.05054 | -0.00168 |
| SBP | 0.54131 | -0.00445 |
| DBP | -0.01456 | -0.00206 |
| LDL-C | -0.00721 | 0.00008804 |
| HDL-C | 0.00345 | 0.00008804 |
| Triglyceride | 0.00805 | 0.00015288 |
| Ln (ACR+1) | 0.00007127 | 0.01284 |
| eGFR | -1.94685 | -0.00903 |
