# Supplementary material for: Hypoglycaemia, chronic kidney disease and death in type 2 diabetes: the Hong Kong diabetes registry
Source: BMC Endocr Disord. 2014 Jun 13;14:48. doi: 10.1186/1472-6823-14-48 (PMC4151079; doi:10.1186/1472-6823-14-48)
Supplement: Additional file 2: Table S2 — Additive interaction of hypoglycaemia events and chronic kidney disease for all-cause death in type 2 diabetes. [file 1472-6823-14-48-S2.doc]

Additional file 2: Table S2 Additive interaction of hypoglycaemia events and chronic kidney disease for all-cause death in type 2 diabetes.

| Measures of additive interaction | Estimate | 95% CI | P values |
| --- | --- | --- | --- |
| Model 1 |  |  |  |
| RERI | 8.04 | 3.69 to 12.39 | 0.0003 |
| AP | 0.50 | 0.35 to 0.64 | <0.0001 |
| Model 2 |  |  |  |
| RERI | 1.70 | 0.45 to 2.96 | 0.0078 |
| AP | 0.39 | 0.20 to 0.59 | 0.0001 |
| Model 3 |  |  |  |
| RERI | 1.46 | 0.31 to 2.61 | 0.0126 |
| AP | 0.37 | 0.17 to 0.58 | 0.0177 |

Abbreviations: RERI, Relative excess risk due to interaction; AP, Attributable proportion due to interaction; * statistically significant with RERI>0 or AP>0 indicating additive interaction;

Models 1, 2 and 3 were adjusted for the same groups of covariates as in Table 2.
